# Selective dorsal neurotomy in the treatment of premature ejaculation: A protocol for systematic review and meta-analysis

**DOI:** 10.1097/MD.0000000000021866

**Published:** 2020-08-21

**Authors:** Guangsen Li, Degui Chang, Di’ang Chen, Peihai Zhang, Yaodong You, Xiaopeng Huang, Jian Cai

**Affiliations:** Hospital of Chengdu University of Traditional Chinese Medicine, Chengdu, Sichuan Province, China.

**Keywords:** dorsal penile neurotomy, premature ejaculation, protocol, surgery

## Abstract

**Introduction:**

Premature ejaculation (PE) affects 8% to 30% of adult men worldwide. Recently, the incidence of PE is on the rise. A series of prior studies suggested that the incidence of PE is related to various biological factors as low testosterone, low serum vitamin D, diabetes, lower urinary tract symptoms, and other psychological factors. At present, the major treatments include selective serotonin reuptake inhibitors antidepressants (dapoxetine, paroxetine), topical anesthetics, phosphodiesterase-5 inhibitor, circumcision, and selective dorsal neurotomy (SDN). The previous study found that SDN is effective for PE.

**Methods and analysis:**

The electronic databases of MEDLINE, PubMed, Web of Science, EMBASE, Cochrane Library, Clinicaltrials. org, China National Knowledge Infrastructure Database (CNKI), Wan fang Database, China Biology Medicine Database (CBM), VIP Science Technology Periodical Database, Chinese Clinical Trial Registry will be retrieved. All the randomized controlled trials of selective dorsal penile neurotomy for patients with PE will be included. The outcome includes intravaginal ejaculation latency time and Chinese Index of Sexual Function for Premature Ejaculation-5. We will conduct this study strictly according to the Cochrane Handbook for Systematic Reviews of Interventions.

**Results:**

The present study is a protocol for systematic review and meta-analysis without results, and data analysis will be carried out after the protocol. We will share our findings on June 30th of 2021.

**Conclusion:**

SDN can effectively prolong IELT, but its efficacy has not been assessed scientifically and systematically. To address this limitation, this study will inspect the efficacy and safety of the SDN treatment in patients with PE.

**Ethics and dissemination:**

Formal ethical approval is not required in this protocol. We will collect and analyze data based on published studies, and since there are no patients involved in this study, individual privacy will not be under concerns. The results of this review will be disseminated to peer-reviewed journals or submit to related conferences.

**Protocol registration number:**

INPLASY202070084

## 1 Introduction

There is very limited literature on the incidence and prevalence of sexual dysfunction. Premature ejaculation (PE), as the substantial body of sexual dysfunction, affects 8% to 30% of adult men worldwide^[1]^ according to 1 US study in 1992. Australian Catholic University reported that the prevalence of PE in a variety of studies exist substantial variability due to definition of PE is not clear.^[2]^ In 2008, the definition of PE is considered by the International Society for Sexual Medicine should include rapidity of ejaculation, perceived self-efficacy, and control and negative personal consequences.^[3]^ After this, the group of Benha University revealed that the prevalence of PE was 26.27%^[4]^ in the condition that the definition of PE has been strictly limited. This indicates that the incidence of PE is on the rise. A series of previous studies suggested that the incidence of PE is related to various biological factors as low testosterone, low serum vitamin D, diabetes, lower urinary tract symptoms,^[5–8]^ and other psychological factors.

During the past decades, the treatment of PE has been increasingly more attention. Due to the various pathogenic factors, scholars still argue for the therapy of PE. The previous studies have largely focused on selective serotonin reuptake inhibitors (SSRIs) antidepressants (dapoxetine, paroxetine), topical anesthetics (TAs), phosphodiesterase-5 inhibitor (PDE5i), circumcision, and selective dorsal neurotomy (SDN).^[9–15]^ A study from Ningbo First Hospital has shown that surgery could be a choice for patients who are resistant to medication after comparing the current therapy.^[16]^ The study of West China Hospital indicated that circumcision is unlikely to adversely affect male sexual functions.^[17]^ The next research further demonstrated that circumcision does not have an effect on PE.^[10]^ The previous study of Moscow found that compared with circumcision SDN is more effective.^[18]^ With the rapid emergence of SDN,^[19]^ the patients who are resistant to medication may get a new kind of treatment.

In recent literature, the effectiveness of SDN obtains several agreements. However, there still lacks adequate evidence to demonstrate the effectiveness of SDN is better than others. The systematic review will analyze and evaluate clinical randomized controlled trials (RCTs) in patients with PE by the method of evidence-based medicine. The purpose of this study is to assess the effectiveness and safety of SDN and provide advice for clinicians.

## 2 Objectives

The purpose of this study is to further evaluate the effectiveness and safety of selective dorsal penile neurotomy in the treatment of PE. The results will provide urologists and andrologists with clinical surgery decisions.

## 3 Methods

The protocol was registered on the International Platform of Registered Systematic Review and Meta-analysis Protocols (registration number: INPLASY202070084) which could be available on https://inplasy.com. The content refers to the statement of the Preferred Reporting Items for Systematic Review and Meta-Analysis Protocols (PRISMA-P) checklist.^[20]^

### 3.1 Eligibility criteria

The inclusion and exclusion criteria are as follows.

#### 3.1.1 Types of Studies

All the RCTs of selective dorsal penile neurotomy for patients with PE will be included without publication status restriction or writing language letters to editors, review articles, case reports, conference abstracts, cross-sectional studies, and all observational studies will be excluded.

#### 3.1.2 Participants

Inclusion criteria:

- Patients who have regular sexual life for >3 months with the fixed sexual partner before the operation, clinically diagnosed as premature ejaculation (≥18 years’ old).

Exclusion criteria:

- Patients who have used antidepressants, topical anesthetics, and other drugs to treat premature ejaculation within 3 months.
- Patients with a history of congenital genitourinary abnormalities.
- Patients with any other disease that the decline of testosterone level.
- Patients with other serious diseases which make them could not complete the trial.

#### 3.1.3 Types of interventions and controls

Experimental interventions:

The patients in the treatment group received selective dorsal penile neurotomy (no restriction on the methods of operation and course of treatment).

Control interventions:

The control group could gain a placebo, no treatment, SSRIs antidepressants, TAs, PDE5is, exercise, or guideline-recommended conventional treatment.

#### 3.1.4 Types of outcome measures

Primary outcome:

1) which could assess the time from when the penis is inserted into the vagina until the beginning of ejaculation.

Secondary outcomes:

1. Chinese Index of Sexual Function for Premature Ejaculation-5 scores.
2. Incidence of adverse events.

### 3.2 Search strategy

#### 3.2.1 Data sources

The electronic databases of MEDLINE, PubMed, Web of Science, EMBASE, Cochrane Library, Clinicaltrials. org, China National Knowledge Infrastructure Database (CNKI), Wan fang Database, China Biology Medicine Database (CBM), VIP Science Technology Periodical Database, Chinese Clinical Trial Registry will be retrieved. They will be searched until May 2021 to recognize related studies. The search strategy that will be run in the PubMed and adjusted to fit the other database when necessary is presented in Table 1.

**Table 1:**
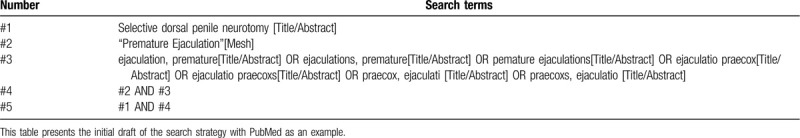
PubMed search strategy.

#### 3.2.2 Other sources of search

Gray literature will be retrieved through Open Grey. Besides, the reference lists of manual review articles will be searched for any possible titles matching the inclusion criteria.

### 3.3 Data extraction, quality, and validation

#### 3.3.1 Study inclusion

**Figure 1:**
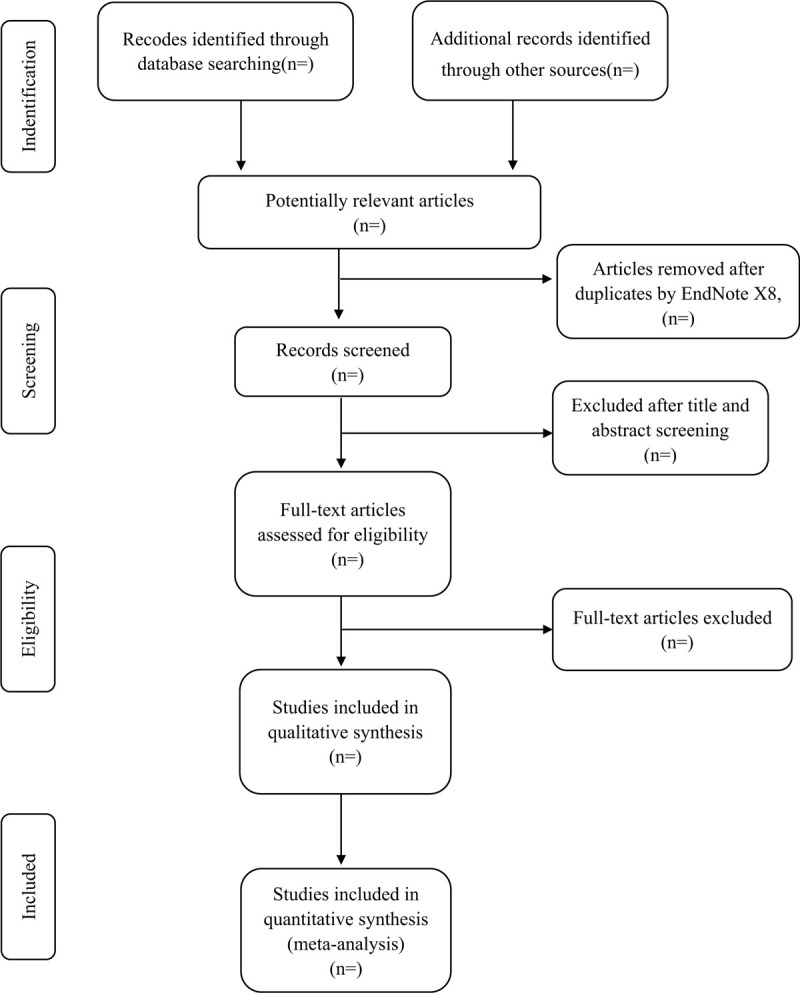
Study selection flow chart.

Importing the literature retrieved to the Endnote X8 and eliminate the duplicate data will be carried out following the above. The software will be used to filter duplicate documents first, and then the studies which do not meet the inclusion criteria will be removed. If the studies appear to meet the inclusion criteria or there is any uncertainty based on the information provided in the title and abstract, full texts will be obtained for further assessment. Further detailed screening and data extraction of the documents will be performed simultaneously by 2 professionally trained reviewers. When the review team cannot confirm the repeated studies, the original study author will be contacted for judgment. Disagreements will be resolved by discussion or taking the expert (GSL) for arbitration. The number and reasons for excluding trials will be recorded in detail. A flow diagram of the study selection is shown in Figure 1.

#### 3.3.2 Data extraction and management

Upon completion of the retrieval, the 2 reviewers will independently read and extract the data from the study. Before the formal process of data extraction, the review group will discuss and a unified data extraction form (an excel spreadsheet) will be produced. The content data will include the following information: title, abstract, first author and corresponding author, the country, the publishing year, publications, participants, demographic characteristics (age, family situation, regional, ethnic, and national), the number of participants, diagnostic criteria, types, intervention, observation index (IELT, CIPE), the results of the study, the incidence of adverse events and type. All disagreement between the 2 reviewers will be decided by consensus or with the participation of a third reviewer. When necessary, we will contact the author via email to request any missing data or clarification. If we cannot obtain the missing data, we will report it in the risk assessment of bias and consider its impact on the analysis of the data.

### 3.4 Risk of bias assessment

Selection bias, detection bias, attrition bias, performance bias, and other bias will be an assessment based on the Cochrane Collaboration Network Risk Assessment Tool. The tool assesses the risk of bias mainly in the following 7 aspects: random sequence generation, allocation concealment, the blinding method for patients, researchers and outcomes assessors, incomplete result data, and selective reports. The risk of bias will be evaluated and checked by 2 review authors. Discrepancies between review authors on the risk of bias will be resolved through discussion with a third review author.

### 3.5 Quantitative data synthesis and statistical methods

#### 3.5.1 Data analysis and synthesis

The RevMan5.3 software will be used to conduct the meta-analysis (if feasible). Descriptive analysis or systematic narrative synthesis will be performed to summarize and explain the characteristics and findings of the included studies and provide the information in the texts and tables. For dichotomous data (eg, effective and ineffective), we will calculate risk ratio (RR) and 95% confidence intervals (CIs). The continuous data will be pooled as mean difference (MD) and 95% CIs.

#### 3.5.2 Investigation of heterogeneity

The Q statistic and *I*^*2*^ statistic of Cochran will be used for testing heterogeneity. If *P* ≤ .10 or *I*^*2*^ ≥ 50%, heterogeneity will be considered significant. At this point, a fixed-effects model (Mantel-Haenzel method for RR and Inverse Variance for MD) will be used for *I*^*2*^ < 50%. A random-effects model (D-I method) will be used when the heterogeneity is still significant after sensitivity analysis and subgroup analysis.

#### 3.5.3 Subgroup analysis

If necessary, we will identify the source of heterogeneity through subgroup analysis and manage the heterogeneity:

1. The site of selective dorsal penile neurotomy.
2. The duration and severity of PE.
3. whether with other sexual dysfunctions.
4. demographic characteristics of the patients: age, marital and family status, region, race.
5. follow-up time.

#### 3.5.4 Sensitivity analysis

Sensitivity analysis will be used to test the reliability and stability of the meta-analysis results, and to assess the source of heterogeneity. We will compare the results before and after by excluding trials with a high risk of bias or eliminating each study individually one study each time and then pooling the remaining studies.

#### 3.5.5 Grading the quality of evidence

The GRADE tool^[21]^ will be applied to judge the quality of evidence in the systematic review. It is consists of risk of bias, consistency, directness, precision, and publication bias. Two independent reviewers will assess these studies. In most cases, disagreements were resolved by discussion between the 2 reviewers. If disagreement remained after discussion, the third reviewer will be consulted before taking the final decision on the disagreements.

#### 3.5.6 Publication bias

Published bias will be measured by the funnel plot. If the result is indistinct, the Begg test and Egger test will be used (by STATA software 11.0).

#### 3.5.7 Reporting of the review

The quality of the manuscript will be standardized by each item of the AMSTAR-2 tool. And the results will be reported following the Preferred Reporting Items for Systematic Reviews and Meta-Analysis (PRISMA) statement.^[22]^

## 4 Discussion

SDN for PE is a microtrauma surgery with less pain. So, it is crucial to make sure whether SDN is a good option for patients. The previous studies have indicated that SDN can effectively prolong IELT; however, its efficacy has not been assessed scientifically and systematically. To address this limitation, this study will inspect the efficacy and safety of the SDN treatment in patients with PE. This review also exists some limitations. The different sites of SDN and the different sizes of PE may induce the heterogeneity.

## Author contributions

**Conceptualization:** Guangsen Li

**Data curation:** Degui Chang, Di’ang Chen

**Formal analysis**: Peihai Zhang, Yaodong You

**Methodology:** Yaodong You, Xiaopeng Huang

**Project administration:** Guangsen Li, Degui Chang, Jian Cai

**Software:** Yaodong You, Xiaopeng Huang

**Supervision:** Peihai Zhang, Yaodong You

**Validation:** Di’ang Chen, Xiaopeng Huang

**Writing – original draft:** Guangsen Li, Degui Chang

**Writing – review & editing:** Guangsen Li, Jian Cai
